# Investigating PM_2.5_ responses to other air pollutants and meteorological factors across multiple temporal scales

**DOI:** 10.1038/s41598-020-72722-z

**Published:** 2020-09-24

**Authors:** Haiyue Fu, Yiting Zhang, Chuan Liao, Liang Mao, Zhaoya Wang, Nana Hong

**Affiliations:** 1 College of Land Management, Nanjing Agricultural University, Nanjing, 210095 China; 2 School of Sustainability, Arizona State University, Tempe, 85281 USA; 3 Department of Geography, University of Florida, Gainesville, 32611 USA

**Keywords:** Environmental impact, Environmental impact

## Abstract

It remains unclear on how PM_2.5_ interacts with other air pollutants and meteorological factors at different temporal scales, while such knowledge is crucial to address the air pollution issue more effectively. In this study, we explored such interaction at various temporal scales, taking the city of Nanjing, China as a case study. The ensemble empirical mode decomposition (EEMD) method was applied to decompose time series data of PM_2.5_, five other air pollutants, and six meteorological factors, as well as their correlations were examined at the daily and monthly scales. The study results show that the original PM_2.5_ concentration significantly exhibited non-linear downward trend, while the decomposed time series of PM_2.5_ concentration by EEMD followed daily and monthly cycles. The temporal pattern of PM_10_, SO_2_ and NO_2_ is synchronous with that of PM_2.5_. At both daily and monthly scales, PM_2.5_ was positively correlated with CO and negatively correlated with 24-h cumulative precipitation. At the daily scale, PM_2.5_ was positively correlated with O_3_, daily maximum and minimum temperature, and negatively correlated with atmospheric pressure, while the correlation pattern was opposite at the monthly scale.

## Introduction

Due to rapid urbanization and industrialization and mounting energy consumption, fine particulates have become a major air pollutant in China’s urban atmosphere^1^. As one of the most important fine particles, PM_2.5_ (particulate matter with aerodynamic diameter less than 2.5 μm) consists of heavy metals, volatile organic compounds, and carbonaceous substances^2^, which could produce detrimental effects on human respiratory system^3^. The World Health Organization (WHO) considers a concentration of PM_2.5_ over 10 μg/m^3^ as hazardous to human health^4^, while this measurement in large cities of China can range from 50 to 125 μg/m^3^^5^. It is estimated that long-term exposure to high level of particulate pollution caused 1.36 million premature deaths per year in China^6^.

To better inform air quality control and build sustainable cities, tremendous efforts have been devoted to understanding potential contributing factors of PM_2.5_ concentration. It is widely recognized that PM_2.5_ are mainly influenced by human activities, natural environment, and atmospheric chemical reactions. Among these three factors, human activities, such as vehicle exhaust emission and industrial production^7,8^, are the dominant factors of PM_2.5_ pollution^9^. Natural environment, such as meteorological conditions (e.g., precipitation and wind speed)^10^, facilitate the transportation and diffusion of PM_2.5_, while atmospheric chemical reactions stimulate the secondary formation of PM_2.5_^11^. All of these factors interact with PM_2.5_ at different spatial and temporal scales, and thus can have varying effects on PM_2.5_ distribution. Tremendous efforts have been paid to understanding such varying effects at multiple spatial scales, such as the national, provincial, and urban cluster scales. In existing literature, the effects of these factors on PM_2.5_ have been primarily investigated at the seasonal scale, while those at the hourly, daily and monthly scales have been understudied. Lack of such knowledge makes it imperative to identify temporally dependent strategies for PM_2.5_ control.

Ensemble Empirical Mode Decomposition (EEMD), a new time-series signal processing method, was recently proposed with the support of signal detection technology^12^. EEMD was designed for non-stationary and nonlinear signal detection and can gradually separate different oscillation or trend components from the original signal^12^. In recent years, EEMD has been adopted by a number of scholars to analyze time series data of PM_2.5_^13–18^, but few of them used it to investigate PM_2.5_ over multiple temporal scales. This article attempts to fill this gap by taking the city of Nanjing, China as the study area. Specifically, the objectives of this study are to: (1) explore the variation of PM_2.5_ at multiple temporal scales, and (2) investigate how PM_2.5_ responds to air pollutants and meteorological factors over different temporal scales. Findings from our research can offer better understanding on the temporal variability of PM_2.5_, and improve regional air quality assessment and air pollution source apportionment.

## Data and methods

### Study area

**Figure 1:**
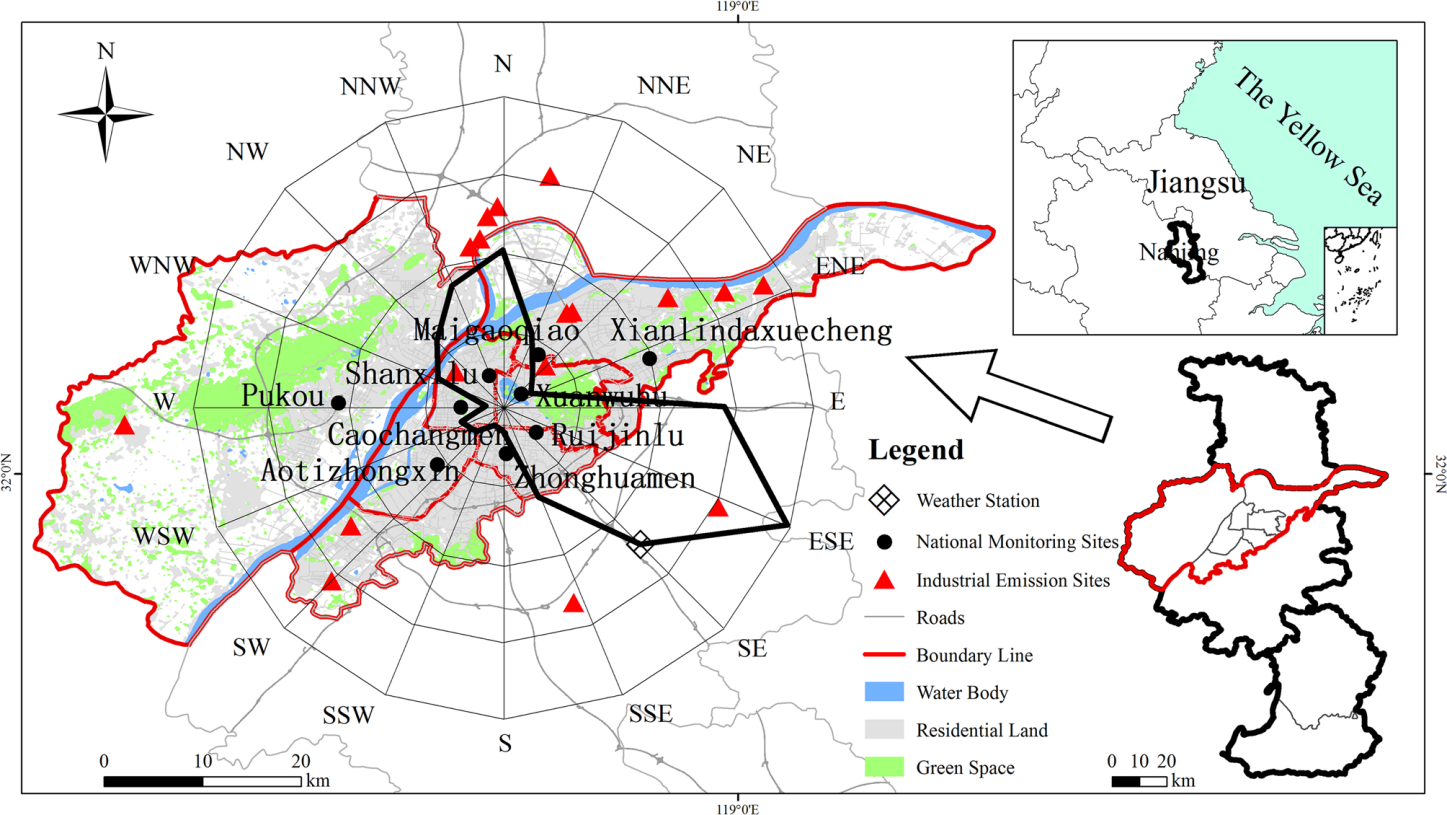
Location of the study area. This figure was created with ArcGIS 10.5 (https://www.esri.com/en-us/arcgis/products/index). Weather Station was retrieved from the National Meteorological Information Center website (https://data.cma.cn/). National Monitoring Sites were retrieved from Jiangsu environmental data public service station (https://218.94.78.75:20001/sjzx/). Industrial Emission Sites were obtained through XGeocoding V2 automatic geocoding after we retrieved the addresses of the polluting enterprise from Jiangsu province key monitoring enterprise self-monitoring information release platform (https://218.94.78.61:8080/newPub/web/home.htm). Roads were retrieved from Wiki world map database (https://www.openstreetmap.org/). Water Body, Residential Land, Green Space were retrieved from the EUlUC-China datasets (https://data.ess.tsinghua.edu.cn/) .The black solid line represents a wind rose that shows the major wind directions.

Nanjing is the capital city of Jiangsu Province in eastern China (Fig. 1). It is situated in the subtropical monsoon climate zone and has four distinct seasons. On the one hand, Nanjing is one of the four garden cities in China, with park green area of 16.01 m^2^ per capita. On the other hand, it is a heavily air-polluted area by PM_2.5_, attributed to the combined effects of large amount of population and on-road vehicles, valley basin landform, numerous polluting enterprises in the suburbs (e.g., petrochemical factories), and the prevailing wind direction of east-south-east (Fig. 1).

### Data

This study acquired three datasets regarding PM_2.5_ concentrations, other air pollutants, and meteorological factors as described below.

### PM_2.5_ and air pollution factors

Besides PM_2.5_, five other major air pollutants monitored in China were included in the analysis, namely, PM_10_, SO_2_, NO_2_, CO and O_3_. We retrieved the hourly concentrations of PM2.5 and five other air pollutants of Nanjing between January 1, 2014 and December 31, 2018 from the Jiangsu environmental data public service station (https://218.94.78.75:20001/sjzx/).

There are nine national monitoring stations in Nanjing (Fig. 1). The hourly concentrations of PM_2.5_ and other five pollutants were derived from the nine stations, and were averaged to indicate daily concentrations of the entire city.

### Meteorological factors

Based on previous studies, wind speed, precipitation, atmospheric pressure, temperature and humidity have shown a strong correlation with PM_2.5_^10,19^, and thus were considered as meteorological factors in this research. For the same time period (January 1, 2014–December 31, 2018), the daily average wind speed (WS), 24-h cumulative precipitation (PR), daily average atmospheric pressure (AP), daily maximum temperature (MaxT), daily minimum temperature (MinT) and daily surface air relative humidity (RH) for the Nanjing city were retrieved from the National Meteorological Information Center website (https://data.cma.cn/) which were reported from the Nanjing weather station, shown in Fig. 1.

### EEMD

EEMD was developed from the Empirical Mode Decomposition (EMD). EMD is a signal analysis method suitable for non-stationary, nonlinear data that can decompose any type of signal in an adaptive manner^20^. The aim of EMD is to decompose raw signals into a set of intrinsic mode functions (IMFs) to better reveal the signal characteristics. The IMFs need to meet the following requirements: (1) the number of extreme and the number of zero crossings must be equal to each other or at most differ by one; (2) at any point, the mean value of the envelope defined by the local maximum and local minimum must be zero.

Although EMD has better adaptability, there is a defect of mode mixing due to the influence of the original signal frequency. Therefore, on the basis of EMD, Wu and Huang proposed a noise-assisted data analysis method-Ensemble Empirical Mode Decomposition (EEMD)^12^. EEMD effectively processes the ‘mode mixing’ issue in EMD by adding white noise to filter IMF components. Meanwhile, EEMD can also complete the significance test that EMD cannot complete by means of perturbation of white noise sets, to obtain the reliability of each IMF^21^. The EEMD algorithm is shortly described as follows:

Denote an original signal as *x*(*t*)*,* where *t* represents time. Then, add a white noise series *w*(*t*) to *x*(*t*) with a certain signal-to-noise ratio and obtain a new signal *X*(*t*):

$$ X\left( t \right) = x\left( t \right) + w\left( t \right) $$

Use EMD to decompose the new signal *X*(*t*) into a number of IMFs:

$$ X\left( t \right) = \sum\limits_{i = 1}^{n} {c_{i} + r_{n} } $$

where *c*_*i*_ represents the *i*th IMF component and *r*_*n*_ denotes the residual.

The above steps are repeated. That is, adding a new white noise series *w*_*j*_(*t*) of the same amplitude to the new signal each time, where *X*_*j*_(*t*) represents the total signal of the *j*th addition of the new white noise, and *c*_*ji*_ represents the *i*th IMF component of the total signal of the *j*th addition of the new white noise, and *r*_*jn*_ represents the residual of the total signal of the *j*th added new white noise:

$$ X_{j} \left( t \right) = \sum\limits_{i = 1}^{n} {c_{ji} } + r_{jn} $$

It is assumed that after adding m-times new white noise series, the decomposed signals satisfy the two requirements of the IMF, and the IMF component *c*_*i*_(*t*) corresponding to the original signal is expressed as:

$$ c_{i} \left( t \right) = \frac{1}{m}\sum\limits_{j = 1}^{m} {c_{ji} \left( t \right)} $$

Similarly, the residual *r*_*n*_(*t*) corresponding to the original signal is expressed as:

$$ r_{n} \left( t \right) = \frac{1}{m}\sum\limits_{j = 1}^{m} {r_{jn} \left( t \right)} $$

The final decomposition result is expressed as:

$$ x\left( t \right) = \sum\limits_{i = 1}^{n} {c_{i} \left( t \right)} + r_{n} \left( t \right) $$

The reliability of each IMF component obtained by EEMD decomposition can be verified by the perturbation of the white noise set. If the IMF energy obtained by the decomposition is above the 95% confidence line compared to the periodic distribution, then the periodic oscillation represented by the IMF component is passed the 5% significance level test, which is the main period of the signal change, also called the strong period; on the contrary, it indicates that the periodic oscillation represented by the IMF component is not very significant, called the weak period.

Relevant to this study, the EEMD method was applied to decompose original time series data of PM_2.5_, other air pollutants (PM_10_, SO_2_, NO_2_, CO and O_3_), and meteorological factors (WS, PR, AP, MaxT, MinT and RH), respectively. Their potential primary components were extracted in order to reveal multi-scale temporal changes. The resulting IMF components showed the cycles, frequencies, and trend that characterize the temporal variation of PM_2.5_, other air pollutant, and meteorological factors. The inherent oscillations of different temporal scales in the original signal (quasi-periodic changes) were also revealed by the IMF components, which reflect the nonlinear relationship between natural driving processes within the atmospheric system and the external human activities, and accurately and succinctly reflect the relationships between PM_2.5_ and both other air pollutants and meteorological data.

## Results

### Descriptive statistics of time series data

Descriptive statistics of PM_2.5_ data with other air pollutants and meteorological factors data of Nanjing from January 1, 2014 to December 31, 2018 are calculated (Table 1). The average PM_2.5_ concentration is 52 μg/m^3^, but the standard deviation is large, indicating the sample data is fluctuant greatly, which has certain research significance.

**Table 1:** Descriptive statistics of air quality and meteorological factors data of Nanjing in 2014–2018.

Air pollution indicator	Unit	Mean	Standard deviation	Meteorological indicator	Unit	Mean	Standard deviation
PM_2.5_	μg/m^3^	52	36	WS	m/s	3	1
PM_10_	μg/m^3^	92	54	PR	mm	28	104
SO_2_	μg/m^3^	17	10	AP	Pa	1013	9
NO_2_	μg/m^3^	47	19	MaxT	°C	21	9
CO	mg/m^3^	0.94	0.35	MinT	°C	13	9
O_3__8h	μg/m^3^	104	51	RH	%	73	14

WS—daily average wind speed, PR—24-h cumulative precipitation, AP—daily average atmospheric pressure, MaxT—daily maximum temperature, MinT—daily minimum temperature, RH—daily surface air relative humidity.

### Temporal variation of PM_2.5_

#### Undecomposed temporal variation of PM_2.5_

**Figure 2:**
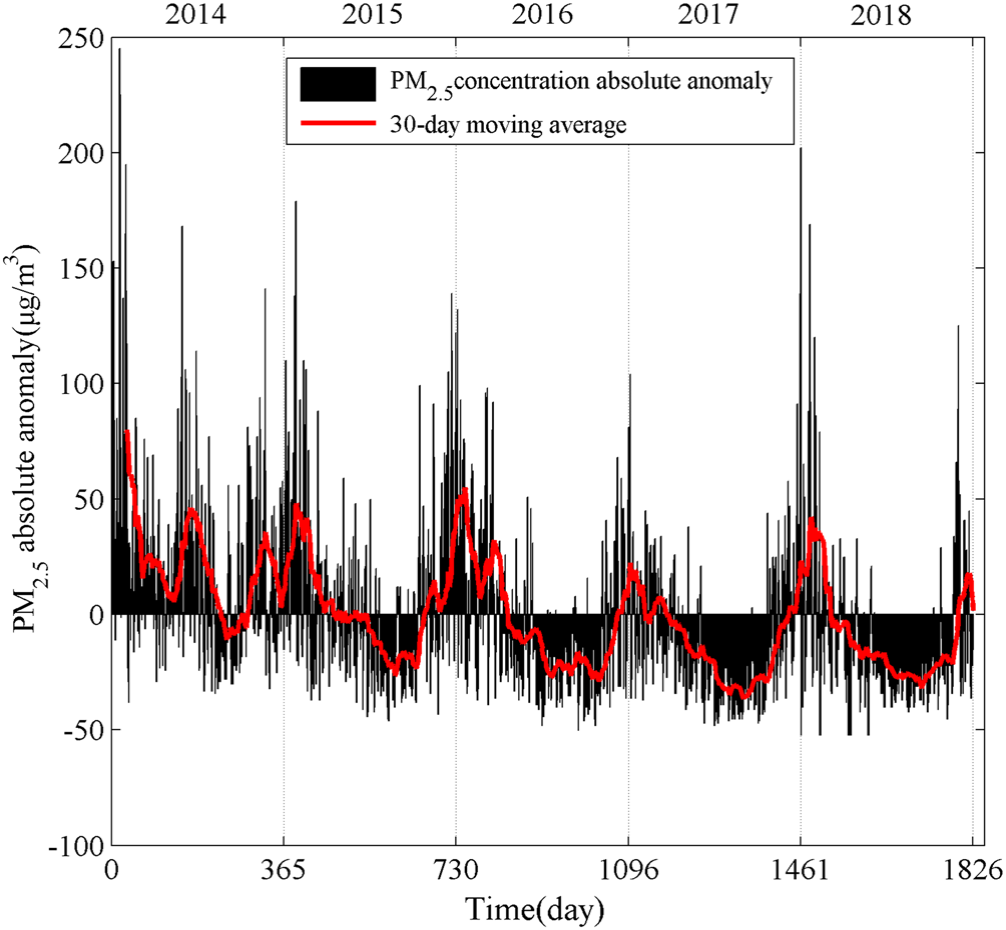
Absolute anomalies in daily average PM_2.5_ concentration in 2014–2018.

Results from the anomaly processing of the average daily PM_2.5_ data suggest that the original daily average PM_2.5_ concentration in Nanjing presented a significant volatility reduction trend during 2014–2018 (Fig. 2). Before December 2017, the PM_2.5_ concentration was relatively higher and presented a weak downward trend, while after that the PM_2.5_ was lower and exhibited the seasonality of “decrease–increase”, i.e., high concentration in spring and winter and low concentration in summer and autumn.

The PM_2.5_ concentration varied dramatically from day to day. The difference between the highest pollution and the lowest was up to 297 μg/m^3^. Meanwhile, the 30-day moving averages curve of PM_2.5_ concentration implied non-linear and non-stationary volatility characteristics of PM_2.5_. Therefore, the EEMD method was needed to further reveal the complex process of PM_2.5_ in Nanjing.

#### Decomposed temporal variation of PM_2.5_

**Figure 3:**
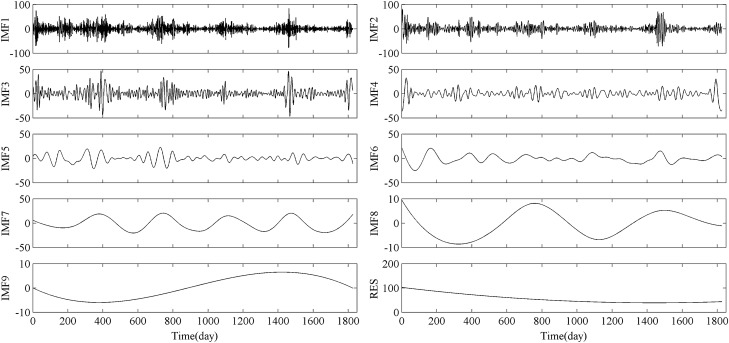
The results of EEMD decomposition of PM_2.5_ daily average concentration of Nanjing in 2014–2018.

The daily average time series of original PM_2.5_ concentration was decomposed by EEMD. For decomposition, the ensemble number was set to 100 and the amplitude of added noise was set to 20% of the standard deviation of the original data. The EEMD analysis produced nine IMF components (IMF1-9) and one trend component (RES), as shown in Fig. 3. Each IMF component a range of frequencies from high (HF, less than a 30-day period) to low (LF, greater than or equal to a 30-day period) at different temporal scales, and the final trend component represents the trend of the original PM_2.5_ data over time.

The significance of each IMF component was tested (Table 2). Except for IMF4, all other components passed the 5% significance level test, indicating that these IMF components contain information with actual physical meaning and the corresponding oscillation periods are the main oscillation period of the PM_2.5_ time series.

**Table 2:** Period, significance level and variance contribution rate of each IMF component of PM_2.5_ concentration.

Time sale	On the inter-day scale					On the inter-month scale				RES
IMF component	IMF1	IMF2	IMF3	IMF4	IMF5	IMF6	IMF7	IMF8	IMF9	
Relief period (days)	3	7	14	27	60	140	406	913	1826	
Confidence level	> 95%	> 95%	> 95%	< 50%	> 95%	> 95%	> 95%	> 95%	> 95%	
Variance contribution rate (%)	19.98	16.33	8.59	4.72	3.96	4.86	11.55	1.96	1.53	26.55

The average daily concentration change of PM_2.5_ has a relatively stable quasi-periodicity (Fig. 3 and Table 2), following the daily circle and the monthly circle. At the daily scale, it exhibits periodic changes of quasi-3 days (IMF1), quasi-7 days (IMF2), quasi-14 days (IMF3) and quasi-27 days (IMF4); At the monthly scale, it exhibits periodic changes of quasi-60 days (IMF5), quasi-140 days (IMF6), quasi-406 days (IMF7), quasi-913 days (IMF8) and quasi-1826 days (IMF9).

According to Table 2, the variance contribution rates of IMF1, IMF2, IMF3, IMF4, IMF5, IMF6, IMF7, IMF8, IMF9 and RES of the daily average concentration of PM_2.5_ in Nanjing are 19.98%, 16.33%, 8.59%, 4.72%, 3.96%, 4.86%, 11.55%, 1.96%, 1.53% and 26.55%, respectively, showing the fact that the contribution rate of high-frequency signal to low-frequency signal of PM_2.5_ concentration gradually decreases. Although the IMF4 component obtained by EEMD decomposition does not pass the significance test, it still contributes to the fluctuation of the original data series.

The influence of PM_2.5_ daily oscillation (IMF1 ~ 4) on the overall variation of PM_2.5_ is 49.61%, while the monthly oscillation (IMF5 ~ 9, RES) is 50.39%, and the contribution of monthly oscillation is slightly higher than that of daily oscillation. Therefore, the daily and monthly scales are two important temporal scales in the PM_2.5_ time series in Nanjing, which is consistent with the temporal of PM_2.5_ that found in other studies as daily, monthly, and annual^22^.

**Figure 4:**
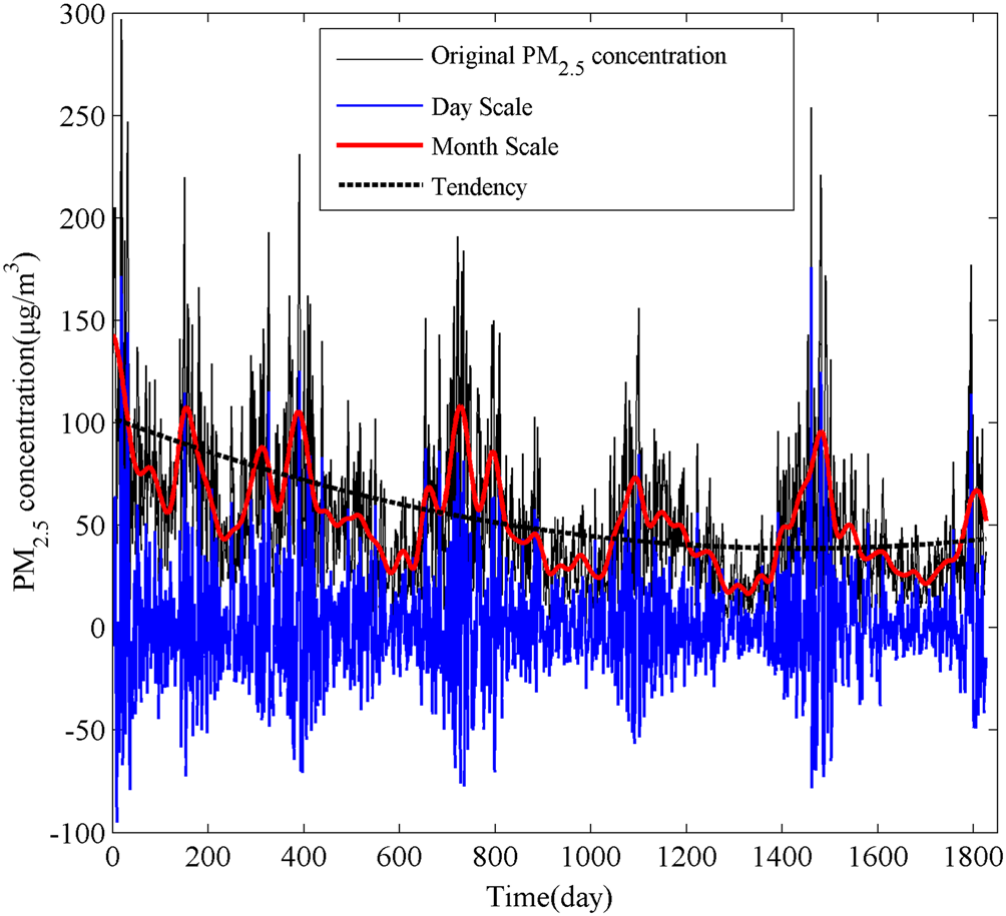
Inter-day and inter-month variations and comparisons with original PM_2.5_ Concentration changes.

The results of PM_2.5_ decomposition in Nanjing were reconstructed at both the daily scale and monthly scale and compared with the original PM_2.5_ concentration and trend (Fig. 4). The inter-day variation was obtained by adding the IMF1, IMF2, IMF3, and IMF4, which can be considered as filtering out large-scale oscillations, while the inter-month variation was obtained by adding the IMF5, IMF6, IMF7, IMF8, IMF9 and RES (including larger time scale fluctuation over the study period).

The reconstructed inter-day variation of PM_2.5_ is basically consistent with the original sequence change trend, which can well show the slight change of PM_2.5_ concentration during the study period, while the reconstructed inter-month variation of PM_2.5_ shows a fluctuation of high concentration in spring and winter and low concentration in summer and autumn throughout the study period, which can effectively reveal the high-low state of PM_2.5_ in Nanjing in different seasons. The inter-day and inter-month fluctuations of PM_2.5_ can reflect the fluctuations of the original PM_2.5_ series from different temporal and have obvious inter-modulation effects as well.

### Response of PM_2.5_ to air pollutants and meteorological fluctuations

#### Undecomposed temporal variation of air pollutants and meteorological factors

**Figure 5:**
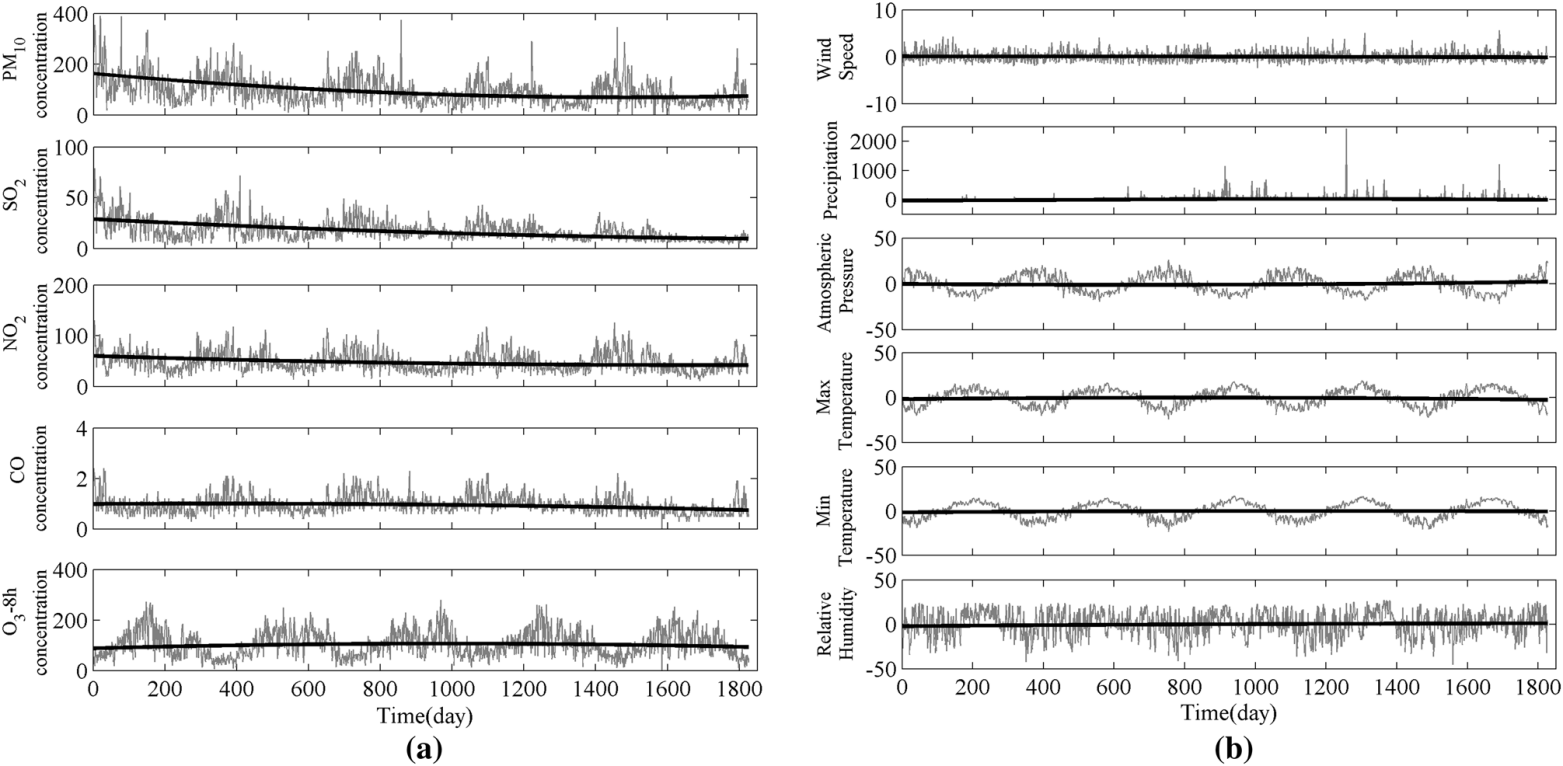
Trends of atmospheric pollutants and meteorological factors in Nanjing from 2014 to 2018.

During the study period, the trends of air pollutants PM_10_, SO_2_, NO_2_, CO, O_3__8h were “decrease–increase”, “progressively decrease”, “decrease–increase”, “increase–decrease” and “increase–decrease”, respectively (Fig. 5a). The trends of meteorological fluctuation—Wind Speed, Precipitation, Air Pressure, MaxT, MinT, and Relative Humidity were “progressively decrease”, “increase–decrease”, “decrease–increase”, “increase–decrease”, “increase–decrease” and “progressively decrease”, respectively (Fig. 5b). The temporal patterns of PM_10_, SO_2_ and NO_2_ are similar to PM_2.5._

The turning points of CO, O_3__8h, Precipitation, Air Pressure, MaxT, and MinT were December 2014, September 2016, January 2017, September 2015, April 2016 and March 2017, respectively, while that of PM_2.5_ was December 2017, indicating that the change of PM_2.5_ had time lags. As important indicators of air quality, CO and O_3_ were inextricably linked to the formation of PM_2.5_. Therefore, PM_2.5_ was found to be responsive to CO, O_3_, PR, AP, MaxT, and MinT.

#### Decomposed temporal variation of air pollutants and meteorological factors

Absolute anomalies in the air pollutants concentration and meteorological factors data were decomposed by EEMD, and the periods were counted (Table 3). The inter-day variation period of air pollutants in Nanjing was highly consistent with PM_2.5_, while at the inter-month scale, the variation period of air pollutants was different from PM_2.5_. Similarly, the inter-day variation period of meteorological factors in Nanjing was generally consistent with PM_2.5._ However, on the inter-month scale, the meteorological factors show larger inter-year variation, which was consistent with the large time scale characteristics of meteorological fluctuation.

**Table 3:** Period of PM_2.5_ concentration, atmospheric pollutants and meteorological factors in Nanjing from 2014 to 2018.

Relief period (days)	PM_2.5_	Atmospheric pollution factor					Meteorological factor					
		PM_10_	SO_2_	NO_2_	CO	O_3__8h	WS	PR	AP	MaxT	MinT	RH
Day scale	3	3*	3*	3*	3*	3*	3*	3*	3*	3*	3*	3*
	7	7*	7*	7*	7*	6	7*	7*	7*	7*	7*	7*
	14	14*	14*	14*	14*	14*	13	12	15	14*	13	15
	27	27*	27*	29	29	29	26	24	29	27*	26	31
Month scale	60	56	57	61	55	59	48	43	55	55	58	63
	140	140*	135	146	140*	146	118	94	406*	365	365	166
	406	406*	406*	406*	332	406*	261	159	522	522	609	365
	913	913*	730	913*	730	1217	406*	365	1826	1217	1217	457
	1826	1826*	1217	1826*	1217	1826*	1217	730	3652	3652	3652	1826*

*The factor period is consistent with the PM_2.5_ concentration change period.

#### Correlation between PM_2.5_ and atmospheric pollutions and meteorological factors

**Figure 6:**
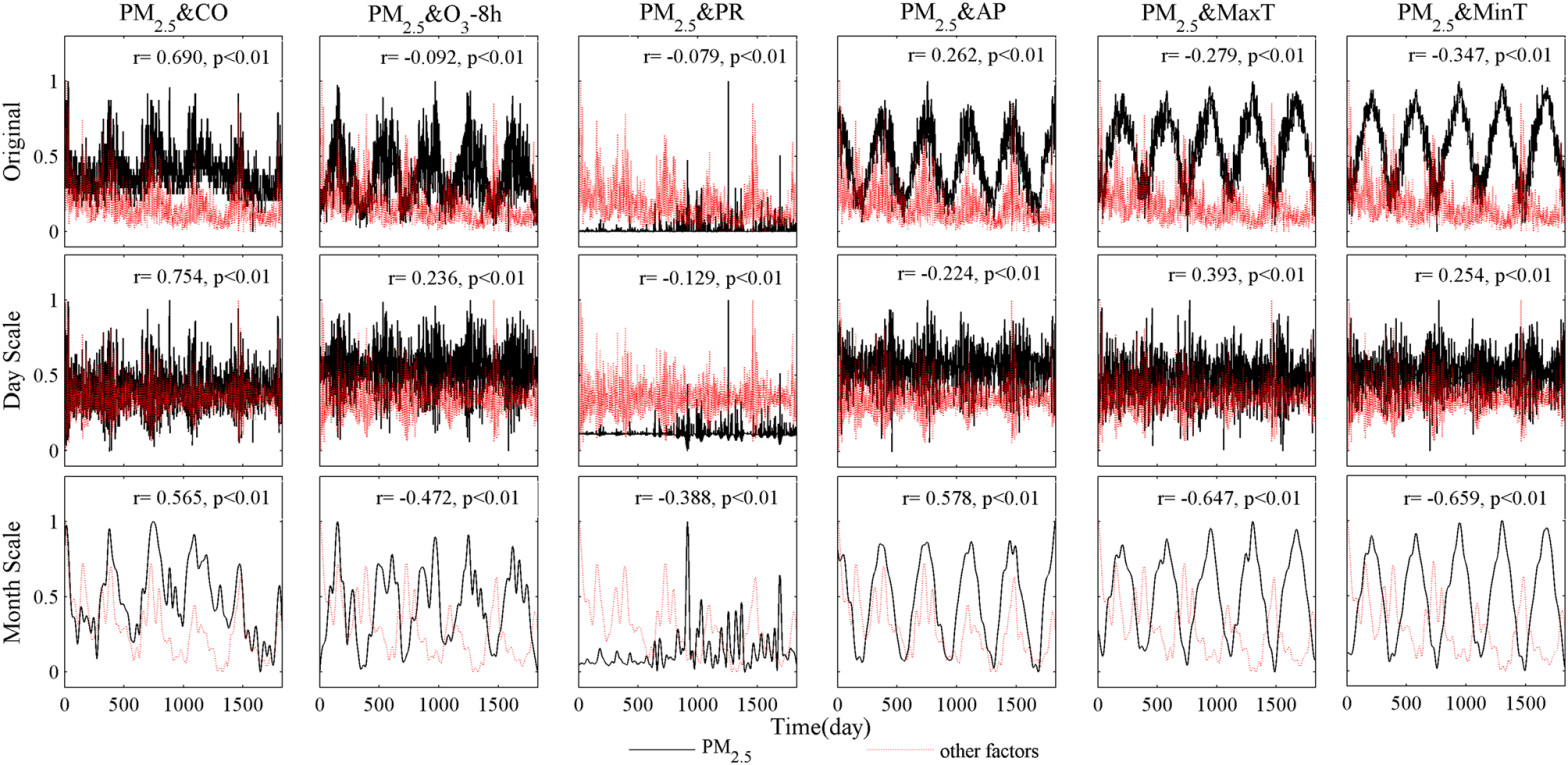
Multi-scale correlations between PM_2.5_ and CO, O_3__8h, PR, AP, MaxT and MinT.

Correlation analysis was conducted between PM_2.5_ and the six metrics at the both reconstructed scale (at the inter-day and the inter-month scale) and original data. As shown in Fig. 6, PM_2.5_ was differently correlated with CO, O_3__8h, PR, AP, MaxT, and MinT at different temporal scales.

The correlation between PM_2.5_ and CO was positively on the daily scale, and the correlation coefficient was slightly higher than that on the monthly scale. The correlation coefficient of the original data was between the two scales. The correlation between PM_2.5_ and O_3__8h was slightly negative when using the original data, but it was positive at the daily scale. At the monthly scale, the correlation was negative and the correlation coefficient was greater than that at the daily scale.

The correlation between PM_2.5_ and PR was negative at both scales and more intense at a broader time scale (inter-month scale). PM_2.5_ and AP were negatively correlated at the inter-day scale, while at the inter-month scale; it was a stronger positive correlation. The original PM_2.5_ showed a significantly negative correlation with MaxT and MinT, and the correlation was positive on the smaller time scale (inter-day scale) but strongly negative at the larger time scale (inter-month scale).

In summary, the responses of PM_2.5_ to O_3__8h, AP, MaxT and MinT were in opposite directions at the daily and monthly scales. The responses were in the same direction for PR and CO at both temporal scales, while the response to PR is more intense at the monthly scale but the response to CO is more obvious at the daily scale. PM_2.5_ has different degrees of significant correlation with other different pollutants and meteorological factors at different temporal, so it has a multi-scale response.

## Discussion

This study contributes to answering the broader question on the anthropogenic and environmental factors that affect the formation of PM_2.5_ pollution. The sources of pollution caused by human activities mainly include fixed sources with fossil fuel combustion and mobile sources with vehicle exhaust emissions. The sulfur and nitrogen oxides produced by them can be directly converted into PM_2.5_. Natural environment mainly involves meteorological factors such as wind speed, precipitation, air pressure, temperature, and humidity, etc. The favorable meteorological conditions conduce to the diffusion and dilution of PM_2.5_ and reduce the risk of pollution.

Human activities are the main cause of PM_2.5_ pollution, and natural environment plays an important role in the aggregation and diffusion of particulate matter. On a short time scale, human activities and environmental actors (especially meteorological conditions) can change dramatically, and thus the contribution of human activities to PM_2.5_ is fluctuant; On a long time scale, human activities have undergone major changes due to the regulation of macroeconomic policies, while overall changes in environmental conditions are small, so the contribution of human activities to PM_2.5_ is stable.

Our findings advance the understanding of how PM_2.5_ responds to the other air pollutants at different temporal scales. PM_10_ is a particle with the size less than 10 μm, which contains PM_2.5_, and its variability is often synchronous with PM_2.5_. SO_2_ and NO_2_ are important precursors in the formation of PM_2.5_, and their trends are similar to PM_2.5_. CO is an intermediate product formed during the combustion of carbon-containing fuels, and it is not directly related to PM_2.5_. However, studies have shown that the spatial distribution of CO and particulate matter concentration tends to be the same in most parts of the day^23^, which supports the conclusion in this study that PM_2.5_ has a strong positive correlation with CO. Under the macro-control of low-carbon emissions, the development of a low-carbon economy in Nanjing controls carbon emissions from adjusting industrial structure, improving energy efficiency and increasing ecological land^24^. Carbon management was carried out from both “source” and “sink”, which also effectively reduced the production of CO. Meanwhile, the vigorous development of new energy vehicles in recent years has alleviated carbon emissions from mobile sources. The production of PM_2.5_, which is based on carbon fuel combustion and vehicle exhaust emissions, is effectively controlled. Therefore, low-carbon policies of energy saving, new energy development is conducive to the regulation of PM_2.5_.

O_3_ is a pollutant formed by the conversion of nitrogen oxides under light radiation and suitable meteorological conditions, and it is also not directly related to PM_2.5_. However, studies have shown that the correlation between O_3_ and PM_2.5_ in different seasons is reversed^25,26^, which is similar to the findings in this study. PM_2.5_ and O_3_ showed significant inverse correlations on the temporal of inter-day and inter-month and the correlation coefficient on the inter-month scale is larger. The source of PM_2.5_ and O_3_ is consistent. The high concentration of O_3_ can promote the formation of secondary particles under strong atmospheric oxidation conditions and increase the concentration of PM_2.5_, and high concentration of PM_2.5_ can weaken solar radiation and inhibit the production of O_3_. On the small time scale, source consistency is an important reason for the significant positive correlation between PM_2.5_ and O_3_, while on the large time scale, the rapid advancement of urbanization, industrialization, and motorization, and massive emission of atmospheric active substances are the main reasons for the increase in surface O_3_ concentration^27^. Since the implementation of the “Air Pollution Prevention Action Plan”, the cooperation mechanism for air pollution prevention and control in the three provinces and one city of the Yangtze River Delta has effectively curbed the growth of PM_2.5_, but O_3_ pollution has become increasingly severe, which indicates that the issue of O_3_ pollution should be further addressed in the continued implementation of the ten policies of the atmosphere.

How does PM_2.5_ respond to the meteorological factors at different temporal scales? The dilution effect of wind speed on PM_2.5_ rises at first and then tends to be gentle^28^, and high humidity environment has the effect of agglomerating PM_2.5_^29^. Studies have shown that precipitation contributes to the dilution of PM_2.5_, and PM_2.5_ concentration before and after precipitation has an important effect on its dilution effect^30^. This study shows that the dilution effect of PM_2.5_ is more significant with the increase of time scale, which provides a new idea for the impact of precipitation on the macroscopic time scale of PM_2.5_.

Few studies have investigated the effect of air pressure on PM_2.5_, and this study found that the response of PM_2.5_ to air pressure is inversely correlated at different temporal scales and is more strongly correlated on the large time scale. Under the low-pressure circulation situation, there are more rainy days and the wind direction changes more frequently, which helps the diffusion and dilution of particulate matter; while the high-pressure circulation situation brings more sunny days and the weather system is relatively stable, forcing the particulate matter to be stagnate in the near-surface layer. Hence, the response of PM_2.5_ to air pressure appears to be positive on a large time scale.

This study found that PM_2.5_ and temperature have a significant correlation at the daily scale, meanwhile, the results show that PM_2.5_ and temperature are significantly negative on the monthly scale, in line with the distribution characteristics of PM_2.5_ “winter-high, summer-low” in Nanjing^31,32^.

This study has shown that there exist seasonal variations of the correlation between PM2.5 concentration and meteorological factors^33–35^. Therefore, the multi-temporal scales cannot be ignored when studying the influence of the natural environment (especially meteorological factors) on PM_2.5;_ meanwhile, the multi-temporal scale provides a new perspective for PM_2.5_ research.

## Conclusion

By investigating PM_2.5_ responses to other air pollutants and meteorological factors at across multiple temporal scales, this research generated three major findings. First, the original daily average concentration of PM_2.5_ exhibited a significant downward trend and the periodic law of “decrease–increase”, which had time lags compared with other air pollutants (CO, O_3_,) and meteorological fluctuation (PR, AP, MaxT, and MinT). Second, the decomposed temporal variation of PM_2.5_ by EEMD has a relatively stable quasi-periodicity in the inter-day and the inter-month temporal scale, and followed daily circle and monthly circle. Third, the responses of PM_2.5_ to other air pollutants and meteorological factors varied at different temporal scales. The temporal pattern of PM_10_, SO_2_ and NO_2_ is synchronous with that of PM_2.5_. At the daily and monthly scales, PM_2.5_ was positively correlated with CO and negatively correlated with 24-h cumulative precipitation. At the daily scale, PM_2.5_ was positively correlated with O_3_, daily maximum and minimum temperature, and negatively correlated with atmospheric pressure, while the correlation pattern was opposite at the monthly scale.

Such findings help improve our understanding on how PM_2.5_ responds to the changes of other air pollutants and meteorological fluctuation at different temporal scales, which contribute to future regional air quality assessment and source apportionment studies. They also suggest that in order to mitigate PM_2.5_ and other air pollution problems, different measures should be devised to target different time scales (especially at inter-day scale and inter-month scale). Especially, the issue of O_3_ increase should be further addressed when we have effectively curbed the growth of PM_2.5_ at a longer temporal scale.

It is important to point out that China has begun to publicize the PM_2.5_ concentration data since December 2013, thus the PM_2.5_ time series of Nanjing selected in this study started on January 1, 2014. With the continuous accumulation of data, longer temporal scales can be considered in future research, especially the inter-annual and inter-decadal variations of PM_2.5_. In addition, the source analysis of the multi-temporal response of PM_2.5_ from two perspectives of human activities (air pollutants) and natural environment (mainly meteorological factors) is mainly realized by qualitative methods so far, and can be further explored by quantitative methods in the future research.
